# The Integration of Prosodic Speech in High Functioning Autism: A Preliminary fMRI Study

**DOI:** 10.1371/journal.pone.0011571

**Published:** 2010-07-13

**Authors:** Isabelle Hesling, Bixente Dilharreguy, Sue Peppé, Marion Amirault, Manuel Bouvard, Michèle Allard

**Affiliations:** 1 UMR-CNRS 5231, Laboratoire d'Imagerie Moléculaire et Fonctionnelle, Université Victor Segalen, Bordeaux, France; 2 Queen Margaret University College, Edinburgh, United Kingdom; 3 CHRS, Charles Perrens, Bordeaux, France; 4 EPHE, Paris, France; University of Groningen, Netherlands

## Abstract

**Background:**

Autism is a neurodevelopmental disorder characterized by a specific triad of symptoms such as abnormalities in social interaction, abnormalities in communication and restricted activities and interests. While verbal autistic subjects may present a correct mastery of the formal aspects of speech, they have difficulties in prosody (music of speech), leading to communication disorders. Few behavioural studies have revealed a prosodic impairment in children with autism, and among the few fMRI studies aiming at assessing the neural network involved in language, none has specifically studied prosodic speech. The aim of the present study was to characterize specific prosodic components such as linguistic prosody (intonation, rhythm and emphasis) and emotional prosody and to correlate them with the neural network underlying them.

**Methodology/Principal Findings:**

We used a behavioural test (Profiling Elements of the Prosodic System, PEPS) and fMRI to characterize prosodic deficits and investigate the neural network underlying prosodic processing. Results revealed the existence of a link between perceptive and productive prosodic deficits for some prosodic components (rhythm, emphasis and affect) in HFA and also revealed that the neural network involved in prosodic speech perception exhibits abnormal activation in the left SMG as compared to controls (activation positively correlated with intonation and emphasis) and an absence of deactivation patterns in regions involved in the default mode.

**Conclusions/Significance:**

These prosodic impairments could not only result from activation patterns abnormalities but also from an inability to adequately use the strategy of the default network inhibition, both mechanisms that have to be considered for decreasing task performance in High Functioning Autism.

## Introduction

Autism is a neurodevelopmental disorder characterized by a specific triad of symptoms such as: abnormalities in social interaction, abnormalities in communication and restricted activities and interests. Communication disorders are considered to be core features of Autism Spectrum Disorders [1]. While verbal autistic subjects may present a correct mastery of the formal aspects of speech, they have difficulties in pragmatics [2], [3]. Pragmatics can be seen as the linguistic conditions of appropriate use of sentences in context: the knowledge of basic speech acts types, such as assertions, questions and commands; the knowledge of all the systems of rules governing “things done with words”, such as congratulations and proclamations; and the knowledge of what is to be included in talk-in interaction pragmatics, such as organization of turn-taking [4]. Pragmatics is essentially conveyed by speech prosody, i.e., the speech musical dimension which is carried by variations of the fundamental frequency (F_0_) and whose perceptual correlate is pitch. Pragmatics includes modifications in pitch, duration and amplitude at the word and the sentence levels. Clinical observations have reported that young children with autism present either a lack of interest in motherese [5], [6] or a marked preference for a synthetic voice resembling motherese [7], which is in favour of a dysfunction in natural speech processing at an early stage of the development. Numbers of studies have reported that autistic subjects, whether children or adults, present prosodic impairment [8]–[12]. Thus, prosodic deficits of every kind pepper autistic speech productions: flat or exaggerated intonation, resulting in inappropriate intonation, abnormalities in rhythm and/or in pitch variations. These productive prosodic dysfunctions appear to persist with age although the formal aspects of speech tend to improve [13]–[16]. Nonetheless, this impairment in prosodic production may stem from a more complex dysfunction concerning prosodic perception, which would be in line with the hypothesis that an abnormality of sensory integration processing would be the core of autism [17].

One test, the Prosody-Voice Screening Profile (PVSP), aims at assessing the speaker's prosody and voice in conversational speech [15]. The only available test assessing both the perceptive and productive prosodic difficulties in English is the Profiling Elements of the Prosodic System (PEPS-C) developed by Peppé and McCann [13]. Different studies using PEPS-C have revealed that subjects with Language-Delayed High-Functioning Autism (LD-HFA) present prosodic deficits, distributed about equally on receptive and expressive prosodic tasks [17] and that the prosodic ability of children with LD-HFA is lower than in children with typical development of the same age from both a productive and perceptive point of view, and somewhat independent of other language skills [6]. These results suggest a perceptive deficit, even if the fact that this perceptive deficit is the cause or the consequence of the productive deficit still remains to be investigated.

Atypical processing of low level perceptual processing has been revealed in the auditory domain [18]. Several studies have reported an enhanced simple low- level processing for pitch discrimination and chord disembedding (spectral processing) [19], [20] whereas other studies have reported that tasks combining spectrally and temporally dynamic, complex material, with complex operations (speech) display a deficit [21], [22]. These findings have been related to the weak central theory which predicts that processing information globally may hamper perceptual functions in autism [23]. However, speech complexity processing by subjects with autism presents a dichotomous picture, since some studies have revealed an enhanced perceptual pitch processing of speech in autism [20]–[24] though other studies have put forward a temporal processing impairment in speech [22] and a lost in the enhanced ability of pitch discrimination in speech [21].

However, while prosodic impairment in autism is beginning to be well documented from a behavioural point of view, little is known about *the neural substrate underlying the integration of prosody*. In typically developing subjects, auditory prosodic processing has revealed the involvement of the frontal, parietal and temporal cortices bilaterally, that is to say the bilateral ventral pathway, the left dorsal pathway and its right counterpart, thus replicating imaging studies in adults [25].

Concerning autism and the basis of prosody, i.e., vocal sounds, an fMRI study of adults with autism and aged-matched controls during passive listening to vocal sounds and non vocal sounds has revealed that the autism group failed to activate bilateral superior temporal sulcus areas, which are considered to be voice-selective areas [26], in response to vocal sounds [27]. Concerning prosody in particular, three studies using cortical-evoked potentials in Asperger have demonstrated deficient encoding of speech and have related this deficit to poor receptive prosody. Kujala and collaborators [28] have reported that adults with Asperger syndrome present a deficit in the processing of pitch variations which would be linked to hypoactivity of the right cerebral hemisphere. Another study has revealed atypical neural responses to affective prosody in children with Asperger and their fathers, especially over the right cerebral hemisphere, and that this impairment can already be seen at low-level information processes [29]. The most recent study using MisMatch Negativity has observed an enhanced response in individuals with Asperger in a constant-feature discrimination for both pitch and vowel stimuli whereas no effect has been revealed when the condition involves deciphering phonemes with pitch variations [21]. The authors have concluded that children with autism lose their advantage in phoneme discrimination when the context of the stimuli is speech-like and requires abstracting invariant speech features from varying input, whereas the discrimination of pitch *per se* is enhanced in autism as compared to controls. A recent study has revealed that children with autism present aberrant, non-direction-specific pitch tracking which could be related to a deficient brainstem encoding of pitch, leading to the hypothesis that abnormalities in pitch processing may stem from an early subcortical processing impairment, which may account for cortical abnormalities [30]. Nevertheless, though no fMRI study has examined the neural correlates of prosodic speech in autism; three fMRI studies have investigated pragmatics in children and adults with autism. They reported increased activation in the right inferior frontal gyrus for subjects with autism as compared to controls when making inferences from discourse [31] or when comprehending pragmatic language [32], which may reflect the higher task demands that subjects with autism faced when interpreting discourse in context. More interestingly, Wang and collaborators [33] have investigated the neural basis of irony comprehension in children and adolescents with High Functioning Autism by differentiating the role of prosody and the role of context. Across all conditions, children with autism presented more activation in prefrontal and temporal regions than control children. More specifically, when only contextual cues were present the right IFG was more activated whereas greater activity was observed in the left Superior Temporal Sulcus and the right temporal pole in children with autism versus children with typical development. The authors have suggested that the greater involvement of the temporal regions may reflect a greater burden for children with autism than for control children when task demands require reliance on prosodic information alone. All together, these studies have revealed that subjects with autism present variations in the involvement of right cerebral cortex as compared to controls, when processing pragmatic, affective prosody or pitch variations in speech.

Along with studies interested in activation patterns, several studies have identified a deficit in the default mode network [34]. When comparing the differences between psychiatric patients such as in autism [34], [35] and controls, fMRI studies have revealed differences in decreased activity in the default mode network between patients and controls. Put another way, when subjects perform a cognitive task, activity in task-related areas increases and default mode activity decreases [36]. Recently, it has been shown that the degree of anticorrelation between activation and deactivation networks is correlated to performance on cognitive tasks [37]. Impairment in the balance between task-dependent activated and task-independent activated networks could be suggested.

Taken together, these data suggest the existence of neural abnormalities underlying language impairment and more particularly *prosodic impairment*. It can thus be hypothesized that an abnormal integration of prosody in speech (requiring both a spectral and a temporal processing) could be at the centre of these deficits. However, the question arises whether this deficit in prosody results from an abnormal neural network functioning, with a hypo or hyper activation of right cortical areas and/or from an altered balance between activated and deactivated networks.

The goal of the present study was therefore to characterize the neural network elicited by the integration of 90-s long connected speech stimuli of high degrees of prosodic information in High Functioning Autism (HFA) using functional Magnetic Resonance Imaging (fMRI). Since speech exists over time, long connected speech stimuli appear to favor a better integration of pitch modulations, since they present much more F_0_ modulations than isolated words or sentences do. The perceptive and productive prosodic abilities were investigated using the PEPS-C so as to assess the prosodic deficits in the HFA group. Results revealed the existence of a link between perceptive and productive prosodic deficits in autism and demonstrates for the first time that the neural network involved in prosodic speech perception exhibits abnormal activation and deactivation.

## Materials and Methods

### Participants

Eight male adults with HFA (mean age 23.38, ±2.10, mean Verbal Intelligence Quotient 89, ±7.89) matched with 8 male controls (mean age 23.05, ±2.02, mean VIQ 128.33, ±4.58) participated in the study after having given their informed written consent in accordance with the guidelines approved by the Ethics Committee of the Bordeaux Medical University. HFA participants were recruited by the Autism Resource Center of Charles Perrens Hospital of Bordeaux and were diagnosed with HFA according to the DSM-IV-R criteria [1] and the ADI-R. They all presented delay in speech onset. Controls were recruited from the community at the University of Bordeaux 2.

No participants had hearing disorders. They had no prior experience of either behavioural or fMRI tasks and were not familiar with the stimulus materials.

### Behavioural study: French adaptation of the English PEPS-C

The PEPS-C [13] was adapted to the French language and culture (Hesling *et al*, in preparation). The French PEPS was implemented with E-prime software (Psychology Software Tools, Pittsburgh, PA), is computerized and lasts 30 minutes.

The procedure aims at evaluating prosodic skills according to a psycholinguistic model [27]. Tasks are at 2 levels: (i) communicative function tasks in which prosody plays an important role (requiring top-down processing, involving meaning) and (ii) form tasks (requiring bottom-up processing, where no meaning is involved). The communicative function tasks are assessed in both receptive and expressive modes whereas the form tasks are assessed in receptive mode in the French version because of the age of the participants since they found the expressive form tasks, i.e., imitation of humming sounds, embarrassing.

- Four communicative functions were transposed so as to assess *both perception and production skills* in French. For each perceptive task, subjects are presented with two images on a computer screen and are required to click on the right image. For the output task, one image is presented and they have to produce what they see. The turn-end task, which involves intonation, aims at assessing the ability to distinguish between a question with rising pitch and a statement with falling pitch. For example, for the input task subjects are required to listen to single words (food items) and decide whether they sound like questions, i.e. if the person on the computer was “asking them if they want some”; or if they sound like statement. For the output task subjects are required to produce this distinction. The chunking task assesses the ability to disambiguate syntactically ambiguous sentences by the use of rhythm and silence. For example, for the input task subjects are required to listen to word groups such as “vingt-quatre, douze” (twenty-four, twelve) versus “vingt, quatre, douze” (twenty, four, twelve) and decide whether it sounds like 2 or 3 figures by clicking on the right image. For the output task one image (for example “thirty-one, twelve”) is presented and they have to produce the distinction.The focus task aims at assessing the ability to evaluate the emphasized word by the use of stress. For example, for the input task, subjects are required to listen to sentences and to identify which item is missing from sentences such as “je voulais du PAIN et des pommes” (I wanted BREAD and apple). For the output task subjects are required to produce this distinction. The affect task assesses the ability to decode the affective state of the speaker as produced using variation in intonation and voice and to produce such an affective state. For example, for the input task subjects are required to listen to one word and decide if the voice likes or does not like the food item by clicking on the right smiley. For the output task, one item is presented with one food item and they have to produce the affect symbolized by the smiley.
- Auditory discrimination abilities are also assessed by 2 form tasks, making it possible to assess whether the subject has the underlying skills required to complete the communicative function tasks. The two form tasks are divided into short item tasks (1 or 2 syllables) and long item tasks (6 or 7 syllables). Short items represent intonation whether long items represent rhythm. The stimuli are laryngograph signals, which sound rather like humming, taken from the recordings of a selection of the four input communicative function tasks.

Each task, whether the 8 receptive and expressive communicative function tasks and the 2 receptive form tasks, includes 18 items with binary responses making it possible to calculate a score. This raw score calculated over 18 is then transformed in percentage. The choice of 18 items is justified by the necessity of having a reasonable number of non-chance scores since the response is binary, as it was done in the PEPS-C [13].

Data were analysed using a Mann-Whitney-U test to assess any differences between the groups for each task. Spearman's correlation test was also done to assess the strength of association between perception and production abilities for each communicative function task in each group. Owing to the heterogeneity of the VIQ in the autistic group, a Spearman's correlation test was done to check if the VIQ interfered with the score of each communicative task.

### fMRI protocol

A 90-s-long prosodic connected speech stimulus dealing with a French story for children, which includes intonation, rhythm, focus and affect prosodic aspects (i.e., the 4 function tasks) was recorded by a trained native speaker in a soundproof room at a 16 bits/44.1 kHz sampling rate. The 90-s-long recording was digitally cut at sentence boundaries to obtain 3 fragments of 30-s-long activation periods. So as to avoid any disturbing noise, the 5 ms of the beginning of each fragment were gradually increased (fade-in process) and the 5 ms of the end of each fragment were gradually decreased (fade-out process).

The fMRI protocol consisted in 3 thirty-second-long stimuli interleaved with 4 fifteen-second-long rest periods, the total length of the procedure being 2 minutes and 50 seconds. Participants were asked to listen to the stimuli while remaining motionless and to keep their eyes closed. The speech stimuli were presented binaurally through headphones specifically designed for use in the scanner (MR Confon, Magdeburg, Germany).

### fMRI acquisition

The MRI data were collected at 1.5 Tesla using an Intera Philips system (Philips Medical System, Best, Netherlands) equipped with an eight-element phased-array head coil. For each subject, a series of 50 functional scans were acquired using a T2*-weighted single shot echo-planar sequence (FOV = 256×256, Matrix = 128×128, TR/TE = 3000/60 ms, Flip angle = 90°, SENSE factor = 2). Each scan included 25 slices (no gap, thickness 4mm) parallel to AC-PC (Anterior Commissure-Posterior Commissure). Three dummy scans were used to reach steady-state magnetization. A high-resolution T1-weighted anatomic scan was also acquired to obtain a morphological reference (25 slices parallel to AC-PC with a resolution of 1×1×4 mm^3^, no gap).

### fMRI debriefing

After the scanning session, the two groups of participants were submitted to a 10 items questionnaire so as to verify they understood the text. Though the autistic group is heterogeneous in VIQ, each subject with autism properly answered the 10 questions, and no significant difference between the two groups was revealed (Student *t*-test, p<0.887).

### Whole brain analyses

All data were analyzed using SPM5 (Statistical Parameter Mapping, Wellcome Department of Imaging Neuroscience, London UK) and MATLAB 7.1 (The Mathworks Inc., Natick, MA, USA) and SPSS 16.0 (SPSS, Chicago).

For each individual subject, the dynamic scans were adjusted for slice timing differences, realigned to the first scan to correct for head movement, normalized to the standard Montreal Neurological Institute space (MNI) and spatially filtered by applying an 8 mm^3^ Gaussian kernel. High-pass filtering (cut off 128s) was performed to remove low frequency artefacts. Then, a general linear model was used to model the data [38]. The functional time series were modeled by a boxcar model convoluted with a canonical hemodynamic response. After estimation of the model parameters, a linear contrast (prosodic speech vs. rest) was built and entered in a 2^nd^ level random effect model. Since the heterogeneity of the VIQ in the autistic group can be confounded, the model was adjusted with VIQ in each group.

#### Activation

A one sample *t*- test was conducted to reveal activated brain areas in each group. A conjunction analysis was performed to determine areas commonly activated in the HFA and control groups [39]. A two sample *t*-test was then run to determine the differences in activation between groups (HFA vs. Controls) for the prosodic listening condition. All data were intensity-thresholded at p<0.01 and cluster size-thresholded, at p<0.05, FDR corrected for multiple comparisons. Anatomical localization was performed using the AAL atlas [28].

#### Deactivation

A one sample *t*- test was conducted to reveal deactivated brain areas in each group. A conjunction analysis was performed to determine areas commonly deactivated in the HFA and control groups [29]. A two sample *t*-test was then run to determine the differences in deactivation between groups (HFA vs. Controls) for the prosodic listening condition. All data were intensity-thresholded at p<0.01 and cluster size-thresholded, at p<0.05, FDR corrected for multiple comparisons. Anatomical localization was performed using the AAL atlas [40].

### ROIs analyses

#### Activation

An ROI analysis was conducted to examine the relation between task performances and activated brain areas extracted from the HFA>controls results. Each ROI was defined as an 8 mm-diameter sphere centered in the coordinates of the peak activated voxels of each activated brain cluster.

A percent BOLD signal change for each ROI was estimated for both groups using MarsBar [41]. Then, a Spearman correlation test was done to assess the strength between BOLD signal and scores of the input tasks.

#### Deactivation

An ROI analysis was conducted to examine the relation between task performances and deactivated areas extracted from the one sample t-test results. Each ROI was defined as an 8 mm-diameter sphere centered in the coordinates of the peak deactivated voxels of each deactivated brain cluster. A percent BOLD signal change for each ROI was estimated for both groups using Marsbar [41]. Then, a Spearman correlation test was done to assess the strength between deactivated areas and scores of the input tasks.

## Results

### Behavioural results

#### French PEPS

**Figure 1:**
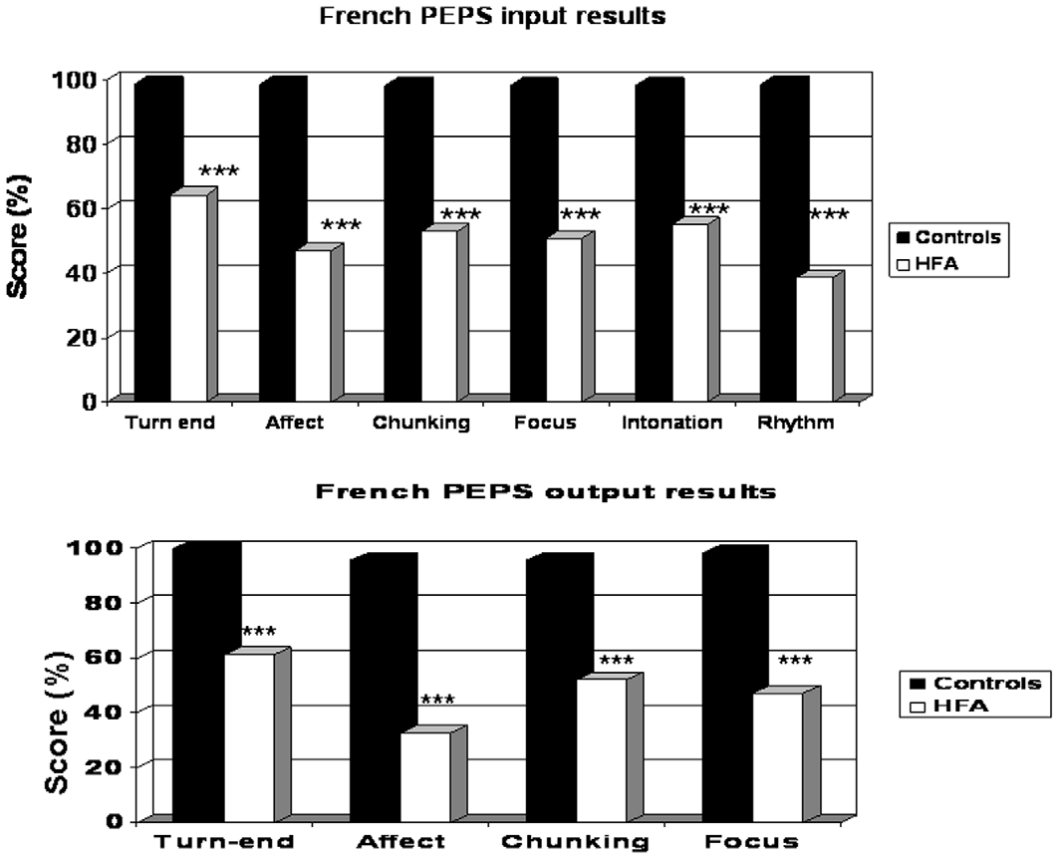
French PEPS input results.

Controls performed all the perceptive and productive tasks at nearly ceiling, though the test cannot be considered as saturated since the score of 100% was only obtained for 2 subtests (expressive Turn-end and expressive chunking). The HFA group's results were significantly lower than those of controls for all the input and output tasks (p<0.001), (Figure 1).

No significant correlation was found between VIQ and any of the communicative tasks using Spearman correlation (Table 1).

**Table 1:** Bivariate correlations between VIQ and receptive and expressive scores for the French PEPS tasks in the HFA and control groups.

PEPS tasks	VIQ HFA	VIQ controls
Function tasks	Rho	
Turn-end input	0.026	0.036
Chunking input	0.021	0.021
Focus input	0.185	0.028
Affect input	0.018	0.018
Turn-end output	0.122	0.022
Chunking output	0.109	0.109
Focus output	0.108	0.108
Affect output	0.073	0.019
Form tasks		
Short Items input	0.112	0.023
Long Items input	0.031	0.015

Rho: Spearman's correlation coefficient.

Statistical analyses using Spearman correlation revealed that 3 communicative tasks (chunking task, p<0.001, Focus task, p<0.01, affect task p<0.01) out of 4 (Turn-end task) presented a significant positive correlation coefficient between perception and production tasks for the HFA group (Table 2). No significant correlation was found for the control group.

**Table 2:** Bivariate correlations between receptive and expressive scores for the French PEPS tasks in the HFA group.

PEPS tasks	HFA	Controls
	Rho	Rho
Turn-end	0.156	0.149
Chunking	0.991**	0.140
Focus	0.869*	0.149
Affect	0.869*	0.140

Rho: Spearman's correlation coefficient.

*: significant at .01 level, *: significant at .05 level.

### Whole-brain analyses

For all the fMRI analyses, the model was adjusted with VIQ.

#### Activation: One sample t test

The bilateral STS and the left cerebellum were activated in both groups whereas the right thalamus was only activated in the autistic group (Table 3).

**Table 3:** Brain activation in HFA and in controls.

Brain areas	K	T_max_	Location (MNI coordinates)		
			x	y	z
*HFA*					
Left MTG	3891	10.09	−48	−24	4
Left cerebellum	232	8.03	−24	−70	−34
Right MTG	3558	17.01	42	−32	2
Right thalamus	301	6.01	0	−6	14
*Controls*					
Left MTG	3136	19.45	−48	−30	−4
Left cerebellum	414	27.38	−10	−82	−34
Right MTG	4002	15.90	52	−18	−22

Note: MTG refers to Superior Temporal Gyrus, ITG to Inferior Temporal Gyrus. One sample *t*-test, thresholded at p<0.01, cluster-sized threshold at p<0.05 FDR-corrected for multiple comparisons, K referring to the cluster size in voxels. The T maxima and MNI coordinates are for the peak activated voxel in each cluster.

#### Activation: Conjunction analysis

**Figure 2:**
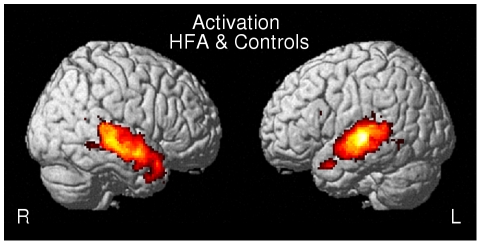
Conjunction map of activation between HFA and controls.

Common activated areas between HFA and controls were observed bilaterally in the middle temporal gyrus (MTG, BA 21), and in the right temporal lobe (MTG, BA 21, ITG, BA38) (Figure 2, Table 4).

**Table 4:** Brain areas commonly activated in HFA and controls.

Brain areas	K	T_max_	Location (MNI coordinates)		
			x	y	z
Left MTG	950	8.45	−56	−26	0
Right MTG	585	6.78	56	−6	−14
Right ITG	50	5.13	46	14	−22

Note: MTG refers to Superior Temporal Gyrus, ITG to Inferior Temporal Gyrus. Conjunction analysis, thresholded at p<0.01, cluster-sized threshold at p<0.05 FDR-corrected for multiple comparisons, K referring to the cluster size in voxels. The T maxima and MNI coordinates are for the peak activated voxel in each cluster.

#### Activation: Two sample t-tests HFA vs. Controls

**Figure 3:**
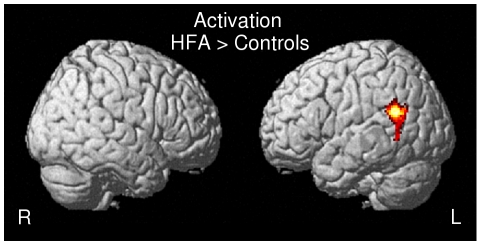
Two sample t tests of activation, HFA>controls.

The HFA group revealed significantly greater activation in the left Supra Marginal Gyrus (SMG) as compared to the control group, whereas no brain area was more activated in the reverse contrast, i.e., Controls>HFA (Figure 3, Table 5).

**Table 5:** HFA >Controls: brain activation.

Brain areas	K	T_max_	Location (MNI coordinates)		
			x	y	z
Left SMG	386	4.38	−44	−52	22

Note: SMG refers to Supra Marginal Gyrus. Two sample *t*-test, thresholded at p<0.01, cluster-sized threshold at p<0.05 FDR-corrected for multiple comparisons, K referring to the cluster size in voxels. The T maxima and MNI coordinates are for the peak activated voxel in each cluster.

#### Deactivation: One sample t test

**Figure 4:**
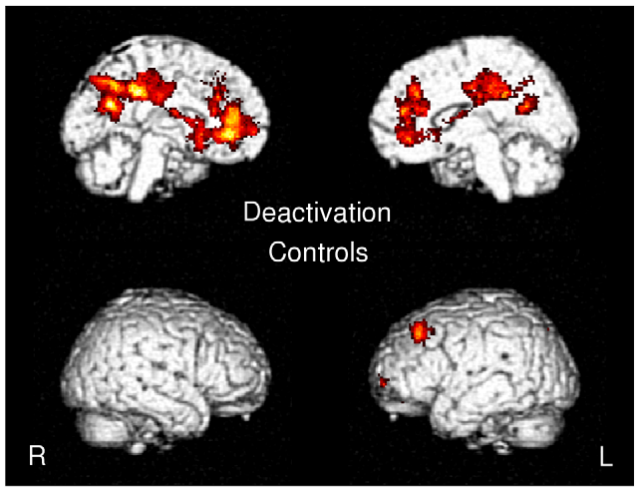
Map of deactivation in controls.

The left precuneus, the right anterior cingulate cortex and the left medial prefrontal cortex deactivated during the prosodic stimulus in the control group, whereas no brain areas were deactivated in the HFA group (Figure 4, Table 6).

**Table 6:** Deactivation in controls.

Brain areas	K	T_max_	Location (MNI coordinates)		
			x	y	z
Left Prec	2759	6.56	−10	−60	24
Left MFG	567	5.60	−32	30	44
Right ACC	2739	5.77	4	26	18

Note: Prec refers to Precuneus, MFG to Middle Frontal Gyrus, ACC to Anterior Cingulate Cortex. One sample *t*-test, hresholded at p<0.01, cluster-sized threshold at p<0.05 FDR-corrected for multiple comparisons, K referring to the cluster size in voxels. The T maxima and MNI coordinates are for the peak activated voxel in each cluster.

#### Deactivation: Conjunction analysis

There were no common deactivated areas between the HFA and the control groups.

#### Deactivation: Two sample t-tests: Controls vs. HFA

The control group revealed significantly greater activation in the left precuneus, the left medial prefrontal cortex and the left middle temporal gyrus as compared to the HFA group, whereas no brain area was more activated in the reverse contrast, i.e., HFA>Controls (Table 7).

**Table 7:** Controls >HFA: brain deactivation.

Brain areas	K	T_max_	Location (MNI coordinates)		
			x	y	z
Left Prec	477	5.34	−12	−44	32
Left MFG	456	5.11	−30	28	42
Left MTG	387	4.38	−44	−52	22

Note: Prec refers to Precuneus, MFG to Middle Frontal Gyrus, MTG to Middle Temporal Gyrus. One sample *t*-test, hresholded at p<0.01, cluster-sized threshold at p<0.05 FDR-corrected for multiple comparisons, K referring to the cluster size in voxels. The T maxima and MNI coordinates are for the peak activated voxel in each cluster.

### Correlations between ROIs analyses and French PEPS

#### Activation

Statistical analyses using Spearman correlation revealed that the left SMG presented a significant positive correlation coefficient with the score of 2 communicative tasks, i.e. the turn-end task (p<0,01) and the focus task (p<0,05) for the natural speech condition in the HFA group (Table 8). No other correlation between cerebral activity and PEPS subtests was found in either group.

**Table 8:** Bivariate correlations between receptive scores of the French PEPS tasks and the left SMG in the HFA group.

PEPS tasks	HFA
Turn-end	0.883**
Chunking	0.153
Focus	0.778*
Affect	0.234

Rho: Spearman's correlation coefficient.

*: significant at .01 level, *: significant at .05 level.

#### Deactivation

Statistical analyses using Spearman correlation revealed that for the control group the left medial prefrontal cortex presented a significant negative correlation coefficient with the score of 3 communicative tasks, i.e., the chunking task (p<0.05), the focus task (p<0.05) and the affect task (p<0.01). The left precuneus presented a significant negative correlation coefficient with the score of 2 communicative tasks, i.e., the turn-end task (p<0.05) and the affect task (p<0.05). The right anterior cingulate cortex presented a significant negative correlation coefficient with the score of 3 communicative tasks, i.e., the chunking task (p<0.05), the focus task (p<0.05) and the affect task (p<0.05).

None of these 3 regions presented a significant correlation with the score of the communicative tasks for the HFA group (Table 9).

**Table 9:** Bivariate correlations between receptive scores of the French PEPS tasks and deactivated areas in both groups.

PEPS tasks	LMPC	Lprecu	RACC
	Rho	Rho	Rho
***Controls***	−0.245	−0.525*	−0.307
Turn-end	−0.419*	−0.291	−0.421*
Chunking	−0.507*	−0.333	−0.599*
Focus	−0.682**	−0.416*	−0.567*
Affect			
***HFA***	−0.231	−0.172	−0.048
Turn-end	−0.152	−0.129	−0.146
Chunking	−0.079	−0.084	−0.148
Focus	−0.157	−0.018	−0.018
Affect			

LMPC: left medial prefrontal cortex, Lprecu: left precuneus and RACC: right anterior cingulate cortex.

Rho: Spearman's correlation coefficient.

* *significant at .01 level.

*: significant at .05 level.

## Discussion

This experiment revealed the existence of a link between perceptive and productive prosodic deficits in autism and demonstrates, for the first time, that the neural network involved in prosodic speech perception exhibits abnormal activation and deactivation. The French adaptation of the English PEPS-C confirmed that subjects with autism not only present difficulties in the production but also in the perception of speech prosody. Moreover, the magnitude of the deficit between perception and production was found to be linked for the HFA group. The fMRI results revealed that brain mechanisms underlying the processing of the prosodic connected prosodic speech comprehension are supported by a different cerebral network in HFA than in controls, involving the left SMG for the HFA as compared to controls. Moreover, whereas controls deactivated brain regions pertaining to the default mode such as the left precuneus and the left middle frontal gyrus as well as the right anterior cingulate while processing the prosodic connected speech comprehension, the HFA group failed to deactivate these brain areas. These results support the existence of a prosodic perceptive impairment in autism.

The French PEPS made it possible to assess significant prosodic differences between the control group and the HFA group, the latter revealing poorer prosodic abilities in both production and perception tasks. This is in accordance with the different results obtained in the English language by the PEPS-C [9]–[17]. More particularly, the Turn-end task, which consists in differentiating between a question and a statement, involves intonation, i.e., pitch variations. It could be suggested, regarding the significantly lower score obtained by the HFA group, that HFA subjects present difficulties in decoding and producing those pitch variations in speech. However, these results are in contradiction with results from Jarvinen-Pasley and collaborators [24] since they have reported an enhanced ability in auditory pitch processing in speech in autism as compared with controls. One issue can be raised to account for these discrepancies in results: in their study, Jarvinen-Pasley and collaborators asked subjects to listen to sentences with 4 different pitch contours and then to match them with a drawing representing the contour. In fact, in this paradigm, subjects can leave aside semantics and concentrate on pitch variations. In our study, as subjects had to match the listened word with the image, they had to integrate both the signifier (the acoustic representation of the word, i.e., the word they listened to) and the signified (the concept, i.e., the image representing the word), [42]. In fact, though both paradigms require high level processing, it may be hypothesized that Jarvinen-Pasley's paradigm involves more low-level processing though the paradigm in the present study involves more high-level processing, which could explain those surface discrepancies.The Chunking task, which allows for disambiguating lexically ambiguous sentences, mainly based on pauses and silences, was also poorly performed. The Focus task, which consists in emphasizing one word in a sentence, was also more difficult for the HFA group, suggesting a problem with stress. As the affect task requires the 3 acoustic correlates of prosody, namely pitch variations, duration (pauses and silences) and intensity, this task was unsurprisingly less well performed by the HFA group than by the control group. Results obtained in the 4 communicative function tasks may be accounted for by results from the 2 form tasks. In fact, these form tasks make it possible to assess whether the subject has the underlying skills required to complete the communicative function tasks. In the present study, these 2 form tasks were significantly poorly performed by the HFA group. More particularly, the short items discrimination task, which represents the ability to process intonation, i.e., pitch variations, is poorly performed by the HFA group, which can be linked to their poor performance in the Turn end task. The long items discrimination task, which represents the ability to process rhythm, is also poorly performed by the HFA group, which can be linked to their poor performance in the chunking task. However, it can be put forward that as these 2 form tasks do not involve a semantics processing, an enhanced processing in the HFA group as compared to controls could have been expected. One possible explanation would be that as these tasks require both spectral and temporal information processing, subjects with autism encounter difficulties with temporal information processing as supported by some studies revealing an abnormal temporal processing of auditory stimuli in speech [18]–[22]. In summary, both perceptive and productive prosodic skills appear to be impaired in the HFA group. Moreover, the magnitude of the perceptive and productive deficits was revealed to be linked for the chunking, focus and affect tasks in the HFA group. This suggests that perception and production deficits are strongly connected and it can be hypothesized that production depends on perception abilities as regard studies on deaf subjects or on second language learning [43].

Data from the fMRI study contribute to understanding this impairment since the cerebral network underlying the processing of prosodic connected speech present differences between the 2 groups. In controls, the bilateral temporal lobes are found to be activated, which is in accordance with previous data showing that auditory sentence comprehension is associated with involvement of both left and right STG [44]–[47]. However, some studies on auditory prosodic speech perception have revealed whether a right [48]–[52] and/or left [53]–[56] Inferior Frontal Gyrus (IFG) activation, which was not achieved in the present study at the chosen threshold as in other studies [57], [58]. One possible explanation is that the content of the stimulus, though prosodic, was not emotional enough to make subjects rehearse the stimulus.

While no brain area was more recruited for the control group as compared with the HFA group; the reverse contrast, i.e., HFA>controls, revealed greater activation in the left SMG. The left SMG has been revealed to be connected with a part of the inferior frontal gyrus (pars triangularis, F3td) through the arcuate fasciculus [59]. The left SMG is viewed as the starting point of the working memory loop for phonology which then projects frontally [60]. As such, the left SMG can be considered as the phonological store area and would then be a part of the phonological loop postulated by Baddeley [61]. It can thus be suggested that autistic subjects rely more on working memory processes and processes translating from auditory to articulatory representations than controls do in the natural condition [62]. Correlations between the left SMG and the Turn-end and Focus tasks in the HFA group revealed that the more this brain structure is activated, the more accurately the HFA subjects performed the tasks. Controls, in the case of natural speech integration, did not present more activation in the left SMG as compared to HFA, though their scores on the task were nearly at ceiling. It can thus be hypothesized that the HFA group recruit the left SMG as a compensatory phenomenon, which is supported by the idea that prosody could be so troublesome for them that they would be more concentrated on phoneme discrimination, which is part of the literal speech decoding, either to avoid paying attention to prosodic features or to be able to understand the story. A further explanation which may be raised for accounting for this left SMG activation could stem from a right hypoactivation in the HFA group, which is in light with previous cortical evoked potential studies reporting a right hypoactivation in autism [28], [29]. In fact, even if the present results did not reveal any differences in the right STS between controls and the HFA group at the chosen threshold, a less permissive threshold revealed that the right STS is more activated in controls than in the HFA group, which would support the hypoactivation hypothesis. Another complementary explanation comes from results from deactivation. When comparing the differences between autistic patients [34], [35], and controls, fMRI studies have revealed differences in decreased activity in the default network between patients and controls, although these differences were not correlated with task performance. In line with this, in the present study, the control group exhibited deactivation in this default mode network while processing prosodic connected speech comprehension, suggesting that listening to the story leads to inhibition of this network engaged in self-reflective thought [63]. The underlying mechanism of this inhibition seems to be a facilitation of task-specific activations through the suppression of task-irrelevant cortical regions, enabling the subject to focus his attention on the relevant process. This hypothesis is supported by results from correlations between the PEPS scores and the 3 seed deactivated regions (the left precuneus, the right anterior cingulate cortex and the left medial prefrontal cortex). In fact, it can be hypothesized that the more these brain regions deactivate, the better the score, which may reflect the degree to which subjects express the balance between tasks-dependent and tasks-independent networks. With this respect, the inabilities of deactivating the default mode network encountered by the HFA group evidenced here could support, at least in part, a less efficient processing of the relevant information, i.e. the prosodic dimension of speech, in autistic patients. The question arises if this deactivation failure results from abnormal functional interaction between task-dependant and task-independent networks or from a dysfunction of default mode network itself. Even if the first hypothesis cannot be excluded, several functional imaging studies in autism have revealed abnormalities in middle anterior and posterior regions involved in the default mode network during a variety of tasks, either in socioemotional [33]–[64] or non-socioemotional tasks [65], [66].

This preliminary study also has several limitations that need to be taken into account when interpreting the findings. Indeed, the results are based on a relatively small sample of subjects and there is heterogeneity in VIQ in the HFA group, which limits the generalization of the results and makes replication efforts an important step. Even if these limits must be considered, three main points can be raised to run counter to them (i) VIQ scores did not correlate with any of the communicative tasks, (ii) each subject properly answered the 10 items questionnaire, making it possible to state that they understood the text and (iii) VIQ was used as a covariate in all the imaging measures.

In conclusion, this study confirms the existence of perceptive prosodic deficits in autism and demonstrates for the first time that the neural network involved in prosodic speech perception exhibits abnormal activation and deactivation. Future studies should further precise the respective role of task dependant and independent networks and assess the direction of the link between perception and production in autism.
